# Veterinary Legislation in California

**Published:** 1895-01

**Authors:** 


DR. LUTHER MARTIN, an old and respected veterinarian of Philadelphia, died December 19, 1894.

THE veterinarians of California should certainly work shoulder to shoulder in the purpose there to secure legislation looking to the proper recognition of the veterinarian officially and as an associate in the better management and care for the health of the people of that State. The law which has been drawn may require some changes and modifications, but its aims and purposes are such that it should command the hearty support of every true votary of the profession in that State.

The veterinary profession in California has been greatly benefited by the workings of the new law, under which it is necessary for veterinarians in towns and cities of 2000 or more inhabitants to undergo an examination at the hands of a Board of Veterinary Medical Examiners, or submit a diploma from a recognized legally chartered veterinary college. Many of the worst empirics have been driven out of the large cities, and others have found it necessary to enter upon other vocations for which we have no doubt they were better fitted. The profession there is having the same experience as has been that of the profession in other States: that the greatest enemies to any law looking toward a higher standard for the veterinary profession are among the qualified men, for whom there was no cause or mitigating circumstances why they should not have given their earnest and unqualified support to such movements, which mean much for the general good of the profession. There will be no effort to extend the law to any new districts until such time as there is a stronger growth in number of the veterinary graduates in that State.

